# Beyond the Break: Radiologic Diagnosis and Outcomes of Necrotizing Fasciitis Complicating Orthopedic Fractures

**DOI:** 10.7759/cureus.94721

**Published:** 2025-10-16

**Authors:** Kiranjot Kaur, Muhammad Zain Ul Abidin, Shahmeen Rasul, Ahmed M Mohamed, Karthika Kalissery Biju Chandrasekhar, Shenouda R Shehata Abdelmesih, Jarallah H. J. Alkhazendar, Faiqa Ijaz, Aliaa H Alkhazendar, Riyan Khalid, Mohit H Buch

**Affiliations:** 1 Internal Medicine, United States Navy, Great Lakes, USA; 2 Clinical Research, Arizona State University, Tempa, USA; 3 Internal Medicine, Shri B M Patil Medical College, Bijapur, IND; 4 Trauma and Orthopaedics, Luton and Dunstable University Hospital, Luton, GBR; 5 Trauma and Orthopaedics, University Hospitals of Derby and Burton, Burton-on-Trent, GBR; 6 Trauma, University of Gezira, Wad Madani, SDN; 7 Trauma and Orthopaedics, The Hillingdon Hospitals NHS Foundation Trust, London, GBR; 8 Orthopaedics and Traumatology, Royal Gwent Hospital, Newport, GBR; 9 Trauma and Orthopaedics Surgery, East and North Hertfordshire Teaching NHS Trust, Lister Hospital, Stevenage, GBR; 10 Medicine, Fatima Jinnah Medical University, Lahore, Lahore, PAK; 11 Surgery, Islamic University of Gaza, Gaza, PSE; 12 Internal Medicine, Jinnah Postgraduate Medical Centre, Karachi, PAK; 13 Radiology, Indrapuri Imaging Centre, Vadodara, IND

**Keywords:** acute kidney injury, necrotizing fasciitis, orthopedic fractures, outcomes, radiologic diagnosis

## Abstract

Necrotizing fasciitis (NF) is a rapidly progressive infection with high mortality. When NF complicates orthopedic fractures, early recognition is particularly challenging because swelling, erythema, and pain may be misattributed to trauma or postoperative changes. A systematic review was conducted using PubMed, Embase, Scopus, and Cochrane databases up to June 2025 according to Preferred Reporting Items for Systematic reviews and Meta-Analyses 2020 guidelines. Eligible studies included human patients with orthopedic fractures complicated by NF in whom radiologic evaluation and clinical outcomes were reported. Risk of bias was assessed using the Joanna Briggs Institute checklist for case reports. Six studies were included, comprising six individual patients with fracture-associated NF. Radiologic evaluation revealed that plain radiographs occasionally demonstrated subcutaneous gas, while CT consistently identified fascial gas, edema, and fluid tracking. MRI was most sensitive for detecting fascial necrosis, particularly in cases under immobilization casts. Clinical outcomes were poor: four patients died, one underwent limb amputation with subsequent death, and one achieved limb salvage with fracture union. Diabetes, ischemia, and virulent pathogens (e.g., *Aeromonas hydrophila*) were major contributors to adverse prognosis. Reporting of systemic complications, including renal dysfunction, was limited despite their recognized prognostic value. Fracture-associated NF is exceedingly rare but carries devastating outcomes, with mortality in the majority of reported cases. CT and MRI play a critical adjunctive role in diagnosis, but cannot replace urgent surgical exploration. Standardized reporting of systemic complications, especially acute kidney injury, is needed. Multicenter registries are essential to establish more robust diagnostic and prognostic frameworks.

## Introduction and background

Necrotizing fasciitis (NF) is a rapidly progressive infection of the fascia and subcutaneous tissue, characterized by fulminant systemic toxicity and high mortality. Its global incidence is estimated at 0.3 to 5 cases per 100,000 population annually, though this may be an underestimation due to under-reporting in low- and middle-income countries [1]. Mortality rates range from 20% to 40%, despite advances in imaging, antibiotics, and surgical debridement [2]. Predisposing factors include diabetes mellitus, chronic kidney disease, immunosuppression, and peripheral vascular disease. In a cohort analysis, diabetes was present in 44% of NF cases, and renal dysfunction independently predicted mortality [3]. Anatomically, extremity involvement is most frequent, followed by truncal and perineal disease. When NF complicates orthopedic fractures, diagnosis is particularly challenging because early symptoms such as swelling, erythema, and pain are easily misattributed to trauma or postoperative change. This diagnostic ambiguity can result in catastrophic delays [4].

Radiologic evaluation plays a critical role. Plain radiographs may show subcutaneous emphysema but have limited sensitivity. CT offers better detection of fascial gas and fluid tracking, while MRI remains the most sensitive tool for mapping fascial edema and necrosis [5]. Ali et al. (2013) confirmed that MRI is highly valuable for diagnosing NF, with deep intermuscular T2 hyperintensity and fascial thickening serving as key differentiators from cellulitis or myositis. Contrast-enhanced sequences further aid early detection, enabling timely and effective management [6]. Despite MRI’s high sensitivity, it has been emphasized that radiology should complement, not replace, clinical suspicion. Recent prognostic studies highlight that renal impairment, advanced age, and delayed debridement are consistent predictors of mortality [7].

This systematic review aims to synthesize current evidence on radiologic diagnosis and clinical outcomes of NF complicating orthopedic fractures, with special attention paid to anatomical extent, pathophysiology, and renal involvement. By consolidating the limited literature, this review aims to highlight knowledge gaps and provide a foundation for future multicenter investigations.

## Review

Methodology

Search Strategy

This systematic review adhered to the Preferred Reporting Items for Systematic reviews and Meta-Analyses (PRISMA) 2020 guidelines to ensure methodological rigor [8]. A comprehensive search was conducted in four electronic databases: PubMed/MEDLINE, Embase, Scopus, and the Cochrane Library, covering all records published up to June 30, 2025. The strategy combined controlled vocabulary terms (e.g., MeSH and Emtree) with free-text keywords, linked with Boolean operators. For PubMed, the search string included terms for the condition (“necrotizing fasciitis,” “necrotising fasciitis,” “necrotizing soft tissue infection”), the exposure (“fracture,” “orthopedic fracture,” “open fracture,” “internal fixation,” “external fixation,” “plaster cast”), radiologic modalities (“radiography,” “X-ray,” “computed tomography,” “CT,” “magnetic resonance imaging,” “MRI”), and outcomes (“treatment outcome,” “mortality,” “amputation,” “renal failure,” “acute kidney injury”). Similar search syntax was adapted for Embase, Scopus, and Cochrane, using Emtree and title/abstract fields where appropriate. Boolean operators were used to refine the search; for example, (“necrotizing fasciitis” OR “necrotising fasciitis”) AND (fracture OR “orthopedic fracture”) AND (radiography OR CT OR MRI) AND (mortality OR amputation OR renal failure). Searches were limited to human studies and English-language publications. To ensure completeness, reference lists of included studies and relevant reviews were manually screened. Grey literature, such as conference abstracts and editorials, was excluded due to insufficient clinical or radiologic data.

Eligibility Criteria

Eligibility was defined using the PICO framework [9]. The Population (P) included human patients of any age with an orthopedic fracture, such as open or closed, treated operatively or conservatively, who subsequently developed NF at or near the fracture site. The Intervention/Exposure (I) was fracture-associated NF with radiologic evaluation using plain radiography, CT, or MRI. No formal Comparator (C) was required, but studies contrasting NF with other soft tissue infections were considered relevant. The Outcomes (O) of interest included radiologic features, anatomical extent, clinical endpoints such as limb salvage, amputation, or mortality, and systemic complications, including acute kidney injury.

We included original reports (case reports, case series, and observational studies) published in the English language that provided full-text patient-level data, described radiologic findings, and documented clinical outcomes. Exclusions were animal or cadaveric studies, in vitro studies, reviews, editorials, letters, and abstracts lacking outcome data. Reports of NF unrelated to fractures or confined to non-orthopedic contexts (e.g., Fournier’s gangrene) were also excluded, as were duplicate publications without new information. Although inclusion of single-patient reports inherently carries a high risk of bias, this was justified by the extreme rarity of fracture-associated NF, making such evidence the only available basis for synthesis.

Study Selection

Two reviewers independently screened titles/abstracts. Full texts of eligible studies were retrieved and evaluated. Discrepancies were resolved by consensus.

Data Extraction

Data were independently extracted by two reviewers. Information collected included study characteristics (author, year, design), patient demographics, fracture type, radiologic modality and findings, clinical outcomes such as amputation or mortality, and systemic complications, with an emphasis on renal involvement. Any discrepancies in extraction were resolved through discussion to ensure accuracy. Data consistency was further verified by cross-checking across both reviewers. This process minimized errors and maintained methodological rigor.

Risk of Bias Assessment

Risk of bias for the included studies was evaluated using the Joanna Briggs Institute (JBI) checklist for case reports [10]. Each study was assessed for clarity of patient description, diagnostic certainty, outcome reporting, and potential confounding factors. Studies were rated as high risk when reporting was incomplete, comparators were absent, or systemic outcomes were poorly documented. Two reviewers performed independent assessments, and discrepancies were resolved by consensus. This ensured a transparent and standardized evaluation of study quality.

Data Synthesis

Given the rarity of fracture-associated NF, all included studies were case reports, preventing quantitative pooling. A narrative synthesis was therefore undertaken, grouping findings by fracture type, radiologic diagnosis, outcomes, and systemic involvement. Frequencies were described in simple proportions (e.g., deaths, amputations, limb salvage). Radiologic features were compared across modalities to identify consistent diagnostic markers. This qualitative approach highlighted recurring clinical patterns while acknowledging the inherent heterogeneity of single-case evidence.

Results

Study Selection Process

**Figure 1:**
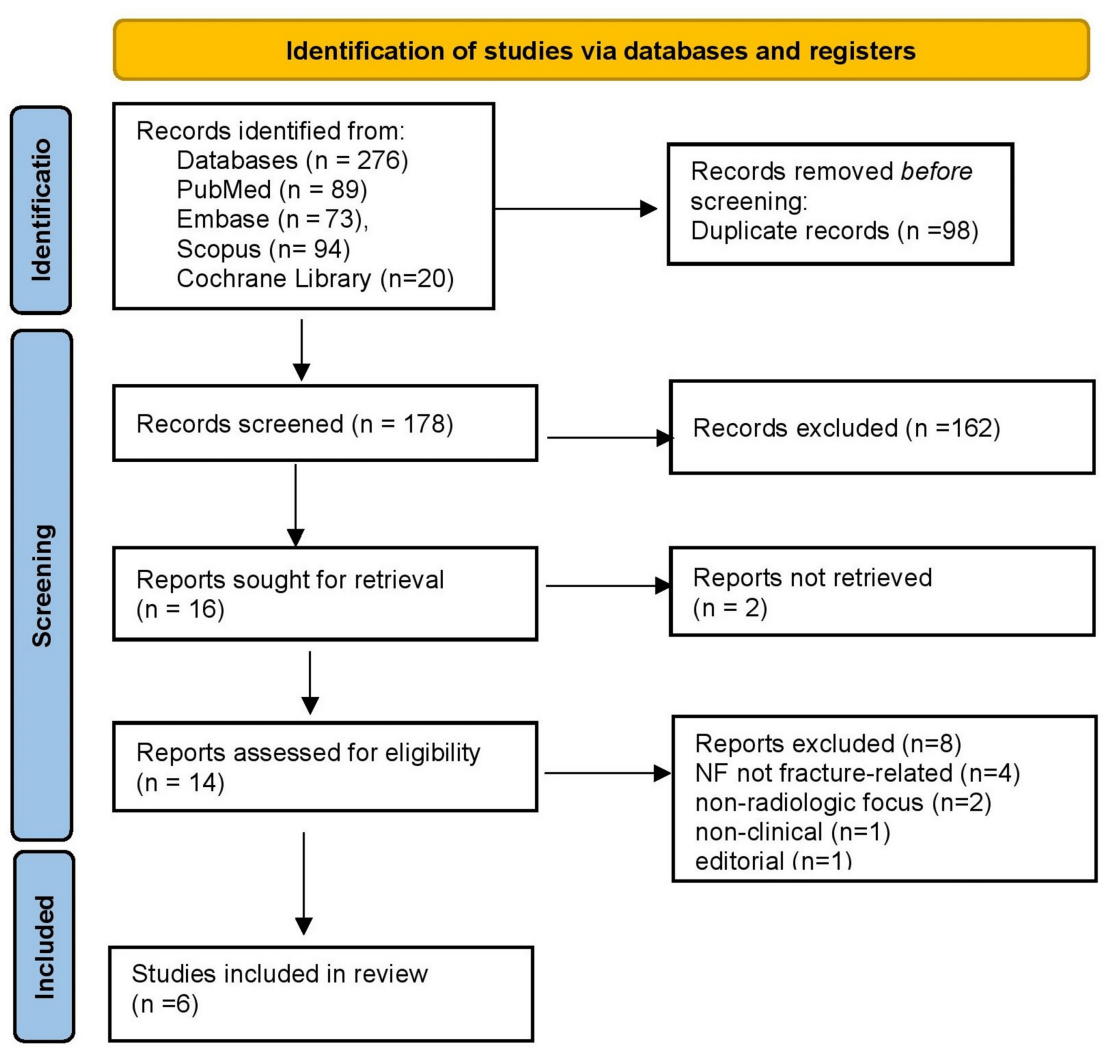
Preferred Reporting Items for Systematic reviews and Meta-Analyses (PRISMA) 2020 flow diagram.

The PRISMA flow diagram is shown in Figure 1. We identified 276 records through database searching: PubMed (n = 89), Embase (n = 73), Scopus (n = 94), and the Cochrane Library (n = 20). After removing 98 duplicate records, 178 unique records remained for screening. Title/Abstract screening excluded 162 records. We sought full texts for 16 reports, of which two were not retrievable. Thus, 14 reports were assessed for eligibility, of which eight were excluded as they did not meet eligibility criteria (e.g., NF not fracture-related, non-radiologic focus, non-clinical, editorial). Six (n = 6) studies met all criteria and were included in the final review.

Characteristics of the Selected Studies

In total, six studies met the inclusion criteria, each representing a single patient with NF complicating an orthopedic fracture. Shang et al. (2017) described an older adult with a tibial shaft fracture treated with minimally invasive plating who developed postoperative NF, ultimately dying from septic shock. Radiographs showed soft-tissue gas, while CT revealed fascial fluid collections [11]. Joomun et al. (2020) reported the case of a 56-year-old diabetic woman with polytrauma who developed incisional NF following hip fracture fixation, extending into the abdominal wall. Despite repeated debridement, the patient succumbed to sepsis, with CT demonstrating fascial thickening and gas [12]. Patil et al. (2021) described the case of a 48-year-old man with a proximal tibia fracture complicated by NF but successfully managed with external fixation and staged surgery, resulting in fracture union and limb salvage at 18 months. Radiographs showed soft tissue swelling confirmed intraoperatively [13]. Hong et al. (2018) presented a fatal case of fulminant NF due to *Aeromonas hydrophila* in an open tibial fracture sustained in an aquatic environment, with CT revealing extensive fascial gas and necrosis [14]. Weidle et al. (2009) detailed the case of a 77-year-old woman who developed NF after a closed distal radius (Colles’) fracture, leading to amputation and death from septic shock. Radiographs demonstrated widespread subcutaneous gas [15]. Finally, Netzer et al. (2009) reported the case of a 43-year-old woman who developed NF beneath a plaster cast following tibial fracture immobilization. She survived after early debridement, with MRI confirming fascial edema that plain radiographs failed to detect [16]. The collective findings from these cases emphasize the devastating outcomes of fracture-related NF, the frequent diagnostic value of CT and MRI, and the influence of comorbidities such as diabetes and ischemia in worsening prognosis (Table 1).

**Table 1:** Characteristics of the selected studies. NF = necrotizing fasciitis; MIPO = minimally invasive plate osteosynthesis; ORIF = open reduction and internal fixation; NPWT = negative-pressure wound therapy; ICU = intensive care unit

Authors and year	Population (P)	Exposure/Condition (I)	Comparator (C)	Outcomes of NF (O)	Pathophysiological findings	Anatomical impact	Radiologic diagnosis
Shang et al., 2017 [11]	Older adult with tibial fracture	Tibial shaft fracture treated with MIPO/ORIF → postoperative NF	None	Death from septic shock and multi-organ failure	Diabetes, immunosuppression, and bacterial toxin-mediated spread	Deep fascia and subcutaneous tissue necrosis; rapid extension	Radiographs showed soft tissue gas; CT revealed fascial fluid and gas
Joomun et al., 2020 [12]	A 56-year-old diabetic female with polytrauma	Hip fracture fixation (ORIF) → incisional NF with abdominal extension	None	Serial debridements, NPWT, and eventual death from sepsis	Idiopathic NF after sterile fixation; systemic inflammatory cascade	Necrosis along the fascial planes from the hip wound to the abdominal wall	CT demonstrated fascial thickening and subcutaneous gas; X-ray was less sensitive
Patil et al., 2021 [13]	A 48-year-old male with a proximal tibia fracture	Proximal tibia fracture complicated by NF, managed with external fixation	None	Limb salvage achieved; fracture united at 18 months	Polymicrobial invasive necrosis; host immune compromise	Extensive skin, fascia, and periosteum with exposed bone	X-rays revealed soft tissue swelling; intraoperative correlation confirmed the diagnosis
Hong et al., 2018 [14]	An adult with a type I open tibial fracture	Open tibial fracture in aquatic setting → fulminant NF (*Aeromonas hydrophila*)	None	Rapid death within days despite debridement and ICU care	Virulent aquatic pathogen; rapid toxin and sepsis	Lower-limb fascia and muscle necrosis extending proximally	CT demonstrated extensive soft tissue gas and fascial involvement
Weidle et al., 2009 [15]	A 77-year-old female with a distal radius (Colles’) fracture	Closed distal radius fracture → upper-limb NF	None	Amputation at the shoulder; death from septic shock	Group A *Streptococcus* septicemia; severe toxin-mediated necrosis	Entire upper-limb fascial compartments destroyed	Radiographs showed extensive soft tissue gas spreading beyond the fracture site
Netzer et al., 2009 [16]	A 43-year-old female with a tibial fracture immobilized in a cast	Tibial shaft fracture in plaster cast → NF under cast	None	Early debridement, survival, and limb salvage with prolonged recovery	Cast pressure/ischemia enabled bacterial invasion; hypoxia promoted necrosis	Fascia and subcutaneous tissues of the leg beneath the cast	Plain radiograph initially inconclusive; MRI confirmed fascial edema and necrosis

Risk of Bias Assessment

All six included studies were single-patient case reports, which inherently carry a high risk of bias. Using the JBI case report checklist, each was rated as high risk due to limitations in generalizability, incomplete outcome reporting, and lack of comparators. The study by Shang et al. (2017) was limited by multiple comorbidities and restricted imaging detail, while Joomun et al. (2020) presented systemic complications but did not report renal outcomes, raising concerns of reporting bias. Patil et al. (2021) demonstrated successful limb salvage, yet selection and publication bias reduced applicability [11-13]. Hong et al. (2018) described a rapidly fatal course with limited imaging documentation, compounded by the unique aquatic exposure [14]. Weidle et al. (2009) reported a lethal case without long-term follow-up, and Netzer et al. (2009) provided mainly narrative inferences about pathophysiology under plaster casting, with limited imaging reproducibility [15,16]. Collectively, the evidence base is constrained by case-level design, underscoring the descriptive rather than generalizable nature of available data (Table 2).

**Table 2:** Risk of bias assessment. JBI = Joanna Briggs Institute

Study	Study design	Risk of bias tool	Risk of bias rating	Justification
Shang et al., 2017 [11]	Case report	JBI case report checklist	High	Single case; outcome influenced by multiple comorbidities; limited imaging detail
Joomun et al., 2020 [12]	Case report	JBI case report checklist	High	Single case; systemic complications; renal outcomes not reported; potential reporting bias
Patil et al., 2021 [13]	Case report	JBI case report checklist	High	Single patient; publication bias toward successful limb salvage; incomplete generalizability
Hong et al., 2018 [14]	Case report	JBI case report checklist	High	Fatal case; very rapid course; limited detailed radiology; ecological exposure unique
Weidle et al., 2009 [15]	Case report	JBI case report checklist	High	Lethal case; no comparator; reporting limited to acute course; missing long-term follow-up
Netzer et al., 2009 [16]	Case report	JBI case report checklist	High	Single case; inference about pathophysiology under cast is narrative; limited imaging reproducibility

Discussion

Fracture-associated NF is a devastating complication where the initial clinical signs, such as swelling, erythema, pain, and wound discharge, are often masked by trauma or postoperative changes. This diagnostic ambiguity frequently results in delayed intervention and poor outcomes. In the six cases identified, outcomes ranged from fulminant systemic sepsis with death to survival with limb salvage. This suggests that fracture-related NF carries an intrinsically worse prognosis due to compounded risks: fracture-related soft-tissue injury, immobilization-induced ischemia, and postoperative inflammatory overlap.

Comparative analysis of these cases highlights key prognostic determinants. Shang et al. [11] and Joomun et al. [12] both reported postoperative NF following internal fixation in diabetic patients, underscoring the role of systemic comorbidities. Patil et al. [13] showed that timely recognition combined with staged external fixation allowed fracture union and functional limb salvage, making it the only long-term favorable outcome. Conversely, Hong et al. [14] illustrated the rapid lethality of *Aeromonas hydrophila* after aquatic exposure, consistent with prior reports that environmental organisms accelerate toxin-mediated necrosis. Weidle et al. [15] described NF after a distal radius fracture, where gas was evident on plain radiographs yet mortality ensued despite amputation, highlighting that radiographic diagnosis does not necessarily alter prognosis if systemic deterioration is advanced. Netzer et al. [16] uniquely described NF beneath a plaster cast, where MRI revealed fascial edema invisible on radiographs, and early debridement secured survival. Together, these cases show that outcomes depend on fracture site, microbial virulence, comorbidities, and above all, the timeliness of surgical intervention.

Radiologic evaluation played a pivotal role in differentiating NF from more benign postoperative complications. Plain radiographs, while occasionally diagnostic when emphysema was pronounced [15], are known to have limited sensitivity (<50%) [17]. CT proved to be the most consistently informative tool, revealing fascial gas, edema, and fluid tracking in multiple cases [11,12,14], and its accessibility in unstable patients aligns with prior evidence of CT’s utility in emergency settings [18]. MRI, as employed by Netzer et al. [16], demonstrated superior sensitivity in detecting fascial necrosis and deep tissue involvement, consistent with prior studies establishing MRI as the gold standard [19]. However, NF remains primarily a clinical diagnosis, with imaging acting as a critical adjunct rather than a replacement for surgical exploration. These findings underscore the necessity of integrating radiologic suspicion with clinical acumen, especially in fracture patients, where inflammation and swelling are expected.

Comorbidities and systemic complications further influenced outcomes. Diabetes was a prominent factor in two cases [11,12], consistent with epidemiological evidence linking diabetes to increased NF mortality. Local ischemia beneath immobilization devices, as in Netzer et al. [16], represents another underrecognized mechanism by which fracture management can predispose to NF. Meanwhile, renal dysfunction, an established independent predictor of poor outcomes, was largely unreported across the included studies. More generally, however, systemic complications are frequent in NF, including sepsis and multi-organ failure remain the leading causes of death, while acute kidney injury occurs in up to 30-40% of patients and independently predicts mortality. Additional complications such as respiratory failure, shock, and disseminated intravascular coagulation further contribute to the high morbidity and mortality associated with the disease [20].

This review is limited by the small number of cases, all being single-patient reports subject to publication bias. Radiologic details, microbiology, and systemic outcomes, especially renal involvement, were inconsistently reported, restricting robust conclusions. Therefore, the findings should be viewed as descriptive rather than generalizable.

## Conclusions

Fracture-associated NF is an exceptionally rare but devastating complication, with six reported patients showing uniformly poor outcomes: four deaths, one fatality after amputation, and only one case of successful fracture union with limb salvage. Imaging supported diagnosis, but never replaced urgent surgery. CT was the most practical tool in unstable patients for detecting fascial gas, edema, and fluid tracking, while MRI provided the highest sensitivity when radiographs were inconclusive or immobilization masked findings. Prognosis was worsened by diabetes, local ischemia (including cast-related pressure), virulent organisms such as *Aeromonas hydrophila*, and delays to debridement. These cases highlight the need for heightened clinical suspicion in fracture patients with disproportionate pain or systemic toxicity, where CT or MRI should accelerate but not delay surgical exploration. Given that all available evidence is limited to single case reports with inconsistent reporting of systemic complications, particularly acute kidney injury, future research should prioritize standardized data collection.
